# The impact of diagnosis-related group-based medical insurance payment model on the prognosis and nursing care of patients undergoing composite trabeculectomy: a retrospective cohort study

**DOI:** 10.3389/fpubh.2025.1518546

**Published:** 2025-05-21

**Authors:** Yu Wang, Yu-yun Zhang, Jie Yan, Tian-bo Ji, Luo-dan Fan, Hai-di Wang, Tong Sun, Dan He

**Affiliations:** ^1^Key Laboratory of Yunnan Province, Yunnan Eye Disease Clinical Medical Center, Yunnan Eye Institute, Affiliated Hospital of Yunnan University, Yunnan University, Kunming, China; ^2^School of Nursing, Kunming Medical University, Kunming, China; ^3^School of Nursing, Yunnan University of Chinese Medicine, Kunming, China

**Keywords:** diagnosis-related group-based medical insurance payment model, composite trabeculectomy, a retrospective cohort study, prognosis, nursing care

## Abstract

**Background:**

The implementation of Diagnosis-Related Groups (DRG) in China’s medical insurance payment system commenced in May 2019, with 30 pilot cities being selected, including Kunming City of Yunnan Province. As part of the nationwide reform of DRG medical insurance payment methods, hospitals are facing increased pressure to decrease average length of stay and improve efficiency within payment cap of different diseases. This study aims to investigate the influence of the DRG-based medical insurance payment reform on the prognosis and nursing care of patients undergoing composite trabeculectomy.

**Methods:**

A retrospective cohort study was conducted on 300 patients who underwent composite trabeculectomy in a Class A tertiary hospital in Yunnan Province between January 1, 2016, and December 31, 2022. Patients were divided into two groups: pre-DRG implementation (DRG group: January 1, 2016 – May 1, 2019) and post-DRG implementation (None-DRG group: May 1, 2019 – December 31, 2022). Data on hospital stay, visual acuity, intraocular pressure, visual field, and retinal nerve fiber layer (RNFL) thickness were collected and analyzed. The relationship between the average length of hospital stay and nursing work patients with composite trabeculae was explored based on the prognostic effect.

**Results:**

The mean length of hospital stay for DRG group is approximately 8 days, compared to approximately 5 days for None-DRG group. The baseline characteristics of the two patient groups were found to be statistically similar (*p* > 0.05). When comparing post-operative follow-up indicators at ≤ 6 months after surgery between DRG group and None-DRG group, including visual acuity (*p* > 0.05), intraocular pressure (*p* > 0.05), field of view (*p* > 0.05), and RNFL (*p* < 0.05), no significant differences were observed from baseline. Similarly, when comparing follow-up indicators after more than 6 months post-surgery between the two groups, visual acuity (*p* > 0.05), intraocular pressure (*p* > 0.05), and field of view (*p* > 0.05) were not significantly different from baseline.

**Conclusion:**

This study represents the first empirical validation demonstrating that the implementation of the Diagnosis-Related Groups (DRG)-based medical insurance reform significantly reduced the mean length of hospital stay for patients undergoing compound trabeculectomy (from 8 days to 5 days). Notably, this reform did not exert a statistically significant impact on key prognostic indicators, including postoperative visual acuity and intraocular pressure (*p* > 0.05). Furthermore, the reform was associated with a marked decrease in hospitalization expenses and nursing costs. These findings offer a robust empirical foundation for refining the DRG policy tailored to ophthalmic care and address a critical gap in the existing literature by providing a comprehensive cost–benefit analysis specific to ophthalmological procedures.

## Introduction

Glaucoma is a spectrum of eye diseases characterized by optic nerve deterioration and corresponding visual field loss, which significantly contributes to irreversible blindness worldwide. Studies suggest that the global prevalence of glaucoma is around 3.5%. It is estimated that by the year 2040, around 111.8 million people will be affected by glaucoma (1). Notably, China has the largest population of glaucoma patients, with a national prevalence rate of 2.5%, and in Yunnan Province, this rate is slightly higher at 2.6% (2). Glaucoma not only significantly impairs patients’ quality of life but also imposes a substantial economic burden on healthcare systems. According to data (1), the annual treatment costs associated with glaucoma in the United States exceed $400 million, while the economic loss due to glaucoma-induced workforce attrition amounts to approximately $20 billion. These figures are extrapolated based on a patient base of 1.4 million glaucoma patients across the nation. Currently, there are no statistical reports available in China regarding the economic losses incurred by glaucoma; however, experts estimate that the direct and indirect economic losses caused by glaucoma in China should be at least five to six times the aforementioned figures. In terms of treatment, surgical intervention is often considered the most effective approach for managing glaucoma. Composite trabeculectomy, an advanced technique, addresses the limitations of traditional trabeculectomy by reducing the risk of early postoperative intraocular hypertension and related complications. As the number of patients increases, so do the medical costs associated with glaucoma, including hospitalization, medication, surgery, and follow-up rehabilitation and nursing care. These costs not only put heavy financial pressure on patients’ families, but also pose a challenge to the sustainability of the healthcare system.

The DRG payment system is widely acknowledged for its sophistication in medical insurance reimbursement, playing a pivotal role in controlling healthcare costs and easing the financial burden on patients (3). The fundamental objective of DRG payment is to enhance efficiency, improve the quality of medical care, and manage costs effectively. By consolidating the grouping and monitoring of patients with similar conditions and streamlining the diagnostic and treatment processes, the system can potentially decrease the average length of hospital stays. This leads to better bed utilization, and a reduction in both the time and financial expenditure associated with patient care (4).

From a health economic perspective, the DRG payment system demonstrates three distinct advantages: By reducing average length of stay (LOS), it directly lowers inpatient costs encompassing bed fees, nursing care, and diagnostic procedures; Through standardized clinical pathways, it mitigates resource wastage associated with overtesting and overtreatment; By optimizing resource allocation, it enhances service efficiency and quality while reducing readmission rates, thereby alleviating long-term financial burdens on healthcare systems.

On December 10, 2018, a significant milestone was marked with the National Healthcare Security Administration’s issuance of a notice detailing the application process for the national pilot program for DRG payment models (5). This initiative, which commenced in May 2019, included Kunming City in Yunnan Province among the 30 pilot cities. The DRG system aimed to allow hospitals to reduce the average length of patient stays within the constraints of illness-specific payment caps, thereby enhancing cost control and alleviating operational burdens. This has led to a reduction in patients’ autonomy regarding the choice of length of hospital stay and the use of medical supplies.

Although previous research has investigated the influence of DRG payment system on hospital costs and average length of stay, a systematic assessment of postoperative prognostic indicators—such as visual acuity, intraocular pressure, and visual field—for ophthalmology-specific procedures (e.g., trabeculectomy) remains limited. Moreover, the majority of existing literature primarily concentrates on medical cost containment, with insufficient exploration into the effects of DRG payment system on nursing workload, nursing quality, and patient care experience. This study pioneers a comprehensive evaluation of the dual impact of DRG payment system on both the prognosis and nursing workload of patients undergoing trabeculectomy, employing a retrospective analysis of a large-scale sample. The findings provide an empirical foundation for optimizing DRG policy in the field of ophthalmology, thereby addressing critical gaps in the current literature.

Thus, the aim of the study was to analyze whether the implementation of the DRG system in Yunnan Province has led to a reduction in the average length of hospital stays for patients undergoing composite trabeculectomy. Additionally, we sought to determine if there were any significant differences in patient prognoses and whether hospitalization and nursing costs had decreased post-DRG implementation. Our hypothesis was that the introduction of the DRG system would indeed shorten average hospital stays without negatively impacting patient prognoses, and would also result in reduced hospitalization and nursing costs.

## Methods

### Patients and data sources

**Figure 1:**
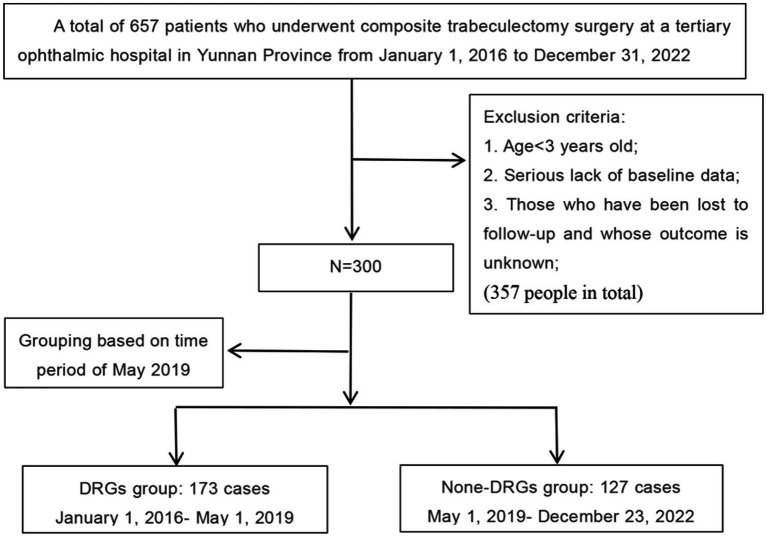
A flowchart illustrating the patient selection process based on the study’s inclusion and exclusion criteria.

This retrospective cohort study, approved by the Medical Ethics Committee of Yunnan University Affiliated Hospital (No. 2024040), was conducted using anonymized data retrieved from both inpatient and outpatient systems. In this study, missing values were managed through multiple imputation for variables with ≤15% missingness and exclusion of records with >20% critical data gaps. The study identified 657 surgical cases that underwent composite trabeculectomy at Yunnan University Affiliated Hospital between January 1, 2016, and December 31, 2022. Participants were excluded if they were less than 3 years old at the time of surgery, had a significant lack of baseline data, or were lost to follow-up. In light of the implementation of the DRG medical insurance reform policy in Yunnan Province starting May 1, 2019, we divided the patients into two groups: DRG group (*n* = 173), encompassing those admitted to the hospital from January 1, 2016, to May 1, 2019, and None-DRG group (*n* = 127), including those admitted from May 1, 2019, to December 31, 2022. The patient selection and grouping process is detailed in Figure 1.

The data extracted from medical records and outpatient systems included demographic information such as name, gender, age, place of birth, and ethnicity, as well as clinical details including diagnosis, preoperative and postoperative visual acuity, preoperative and postoperative intraocular pressure, postoperative RNFL, length of hospital stay, hospitalization expenses, and nursing expenses. Prognostic indicators were established based on follow-up data for visual acuity, intraocular pressure, field of view, and RNFL at ≤ 6 months or > 6 months. An Excel electronic database was established to facilitate statistical analysis.

### Statistical analysis

Continuous variables: mean *±* standard deviation of variables conforming to normal distribution, median and 25 and 75% quartiles of variables conforming to normal variables. Categorical variables: *n* (%). Propensity scores are used for the treatment of confounders. Wilcoxon’s rank-sum test and Fisher’s exact test were used for comparison of baseline data between the two groups. The Wilcoxon rank-sum test was used for comparison between groups A and B. Multivariate *ANOVA* was used for intra-group comparisons.

## Results

**Figure 2:**
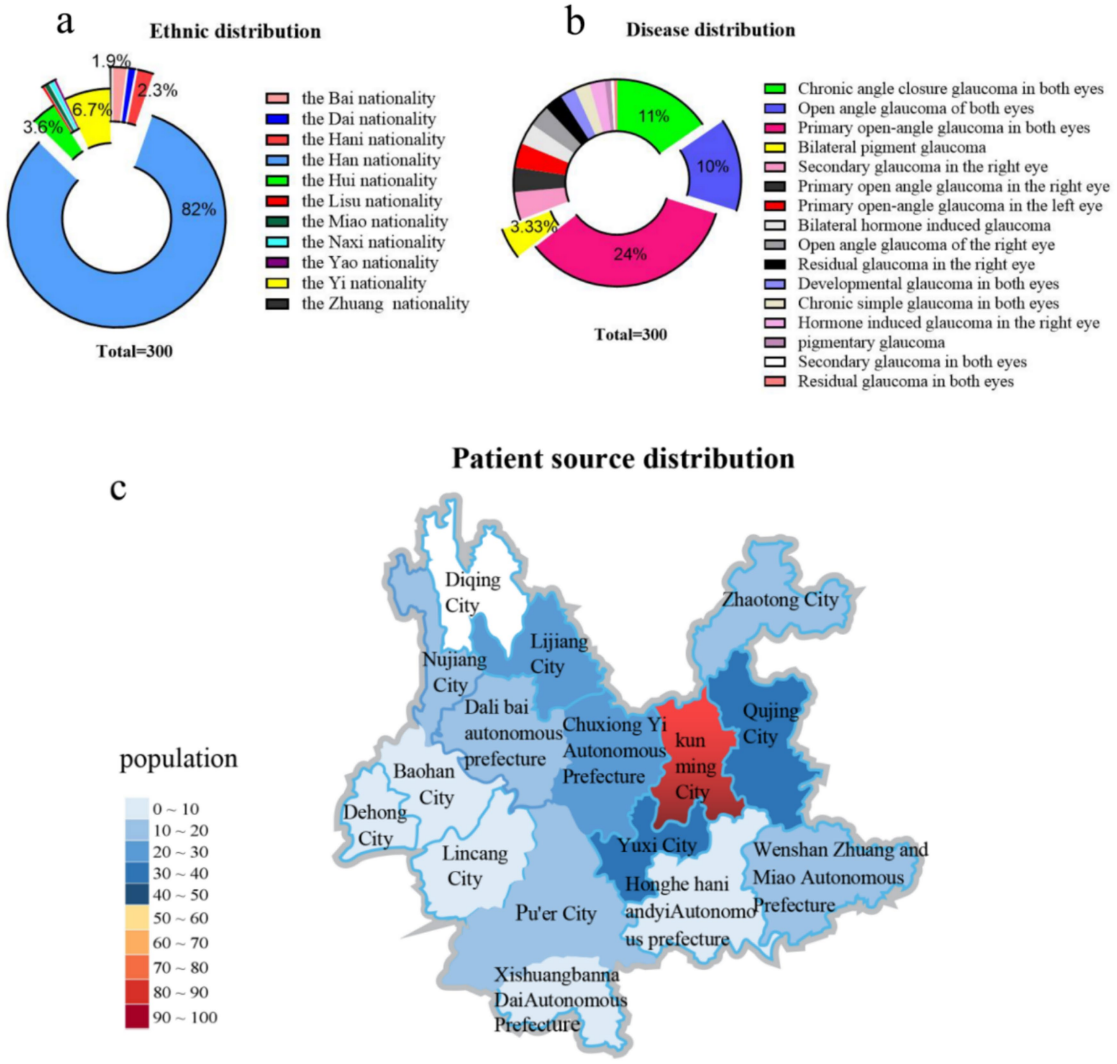
The chart illustrates the distribution characteristics of the 11 ethnic groups included in the dataset, based on the 56 unique ethnic groups recognized in China, with a sample size of 300 individuals. **(a)** The figure shows the distribution characteristics of disease diagnosis names in the pie chart included in the data. The disease diagnosis names in the figure are based on the “Guidelines for the Diagnosis and Treatment of Ophthalmic Diseases (2nd edition).” **(b)** The figure shows the population distribution of patients included in the data from Yunnan Province, China. The “population” example in the figure represents a difference of 10 people in the interval **(c)**.

A total of 300 patients were enrolled in the study. Their demographic and clinical characteristics are detailed in Supplementary Table 1. The majority of the patients were of Han ethnicity (*n* = 246, 82%), followed by Yi (6.67%), Hui (3.6%), Hani (2.3%), and Bai (1.9%). Approximately 31% of the patients lived in Kunming, Yunnan, while the remaining were distributed across other cities. The study cohort included 72 cases of primary open angle glaucoma in both eyes (24%), 32 cases of chronic angle closure glaucoma in both eyes (11%) and 30 cases of open angle glaucoma in both eyes (10%). The median age of the patients was 36 years (interquartile range 25–75: 55 years), with ages ranging from 6 to 89 years. The gender distribution was 209 males (69.67%) and a male to female ratio of approximately 2.3:1. The study cohort was divided into two groups: DRG group (*n* = 173) and None-DRG group (*n* = 127), with DRG group comprising patients admitted between January 1, 2016, and May 1, 2019, and None-DRG group comprising patients admitted between May 1, 2019, and December 31, 2022. The average length of hospital stay in DRG group is about 8 days; whereas for None-DRG group, it was approximately 5 days. The formula for calculating the average length of hospital stay is as follow: Average length of stay = Total bed days of discharged patients/Number of discharged patients during that period (Figure 2).

**Figure 3:**
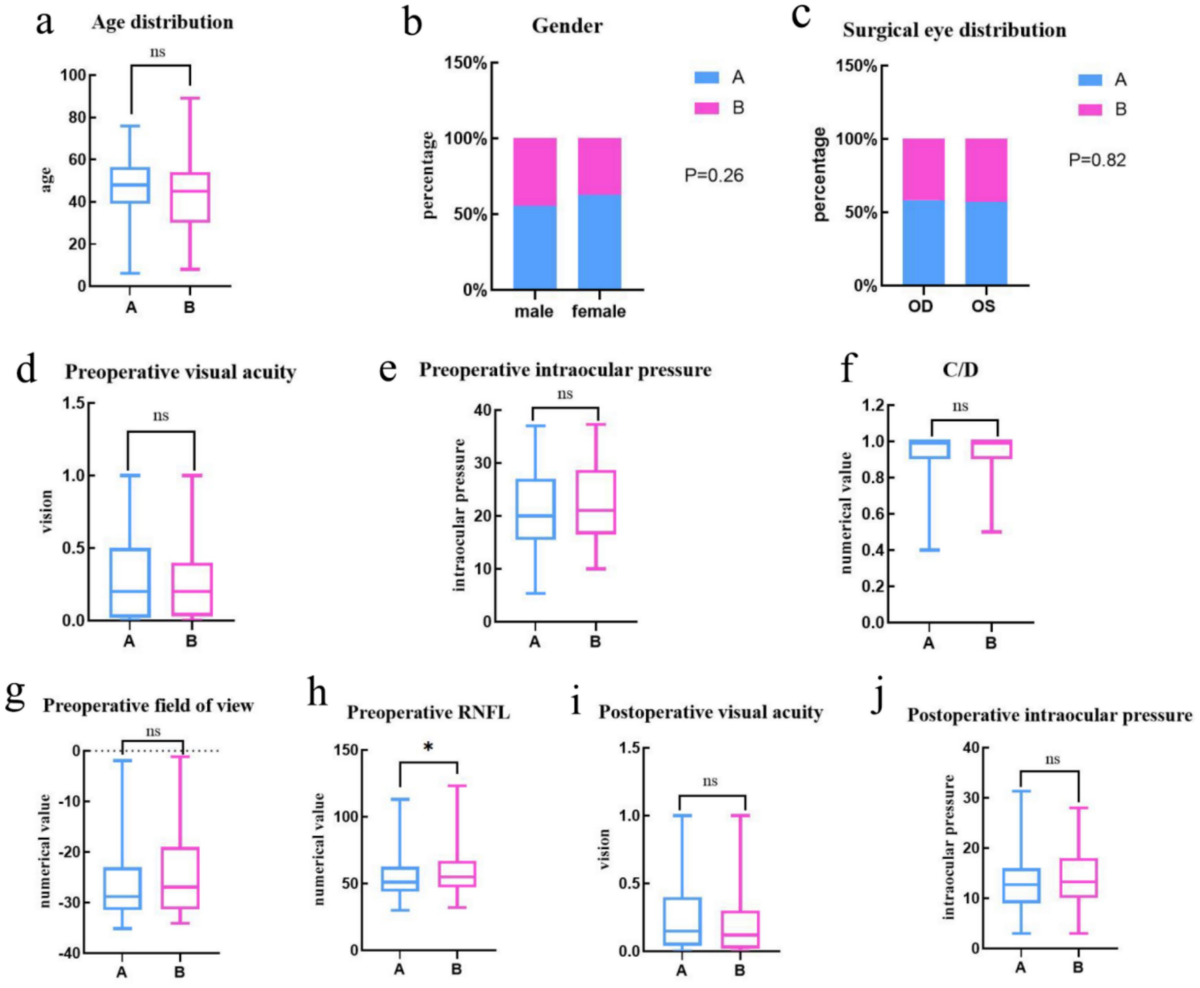
The X-axis represents the DRG group, the None-DRG group, and the Y-axis represents age. **(a)** The Y-axis represents the visual acuity value. **(d,i)** The Y-axis represents intraocular pressure (mmHg) values. **(e,j)** The Y-axis represents the C/D value. **(f)** The Y-axis represents the numerical value (dB) of the field of view. **(g)** The Y-axis represents the RNFL (μm) state. **(h)** The X-axis represents male, female, and the Y-axis represents the sex ratio. **(b)** The X-axis represents the operative eye (OD, OS) and the Y-axis represents the operative-eye ratio. **(c)** Statistical tests: **(a,d–j)** uses the Mann Whitney’s test, **(b,c)** uses the Fisher’s exact test; Significance level: * = *p* < 0.05; “ns” means not significant.

Figure 3 displays the baseline data analysis of the two patient groups, illustrating the age distribution as follows: DRG group had a median age of 44 years (IQR: 29–54), and None-DRG group had a median age of 48 years (interquartile range: 39–56), with no statistically significant difference between the groups (*p*-value = 0.052). The Gender distribution in two groups was as follow: DRG group included 116 males and 57 females, while None-DRG group included 93 males and 34 females, with no statistically significant difference (*p*-value = 0.26). The distribution of surgical eyes between the two groups was also similar: DRG group had 91 cases in the right eye and 82 cases in the left eye, while None-DRG group had 65 cases in the right eye and 62 cases in the left eye, with a *p*-value of 0.82 for both groups. Preoperative visual acuity: 0.2 (0.02, 0.5) in DRG group and 0.2 (0.03, 0.4) in None-DRG group, with a *p*-value of 0.13 for both groups. Preoperative intraocular pressure: 20 (15.5, 30.65) mmHg in DRG group and 21 (16.5, 32.7) mmHg in None-DRG group, with a *p*-value of 0.44 for both groups. The preoperative C/D ratio for both groups A and B was 1.0 (0.9, 1.0), with a *p*-value of 0.97(C/D is the cup-to-disc ratio, which is an important indicator in the diagnosis of glaucoma. Cup-to-disc ratio is the ratio of the diameter of the optic cup to the diameter of the optic disc and is used to evaluate structural changes in the optic nerve head and help diagnose optic nerve diseases such as glaucoma.). The postoperative visual acuity (*p* = 0.052), postoperative intraocular pressure (*p* = 0.10), and preoperative visual field (*p* = 0.13) of the surgical eye all have *p* values greater than 0.05, indicating comparability. The comparison of RFNFL between DRG group and None-DRG group before surgery (*p* = 0.03 < 0.05) showed statistically significant differences.

**Figure 4:**
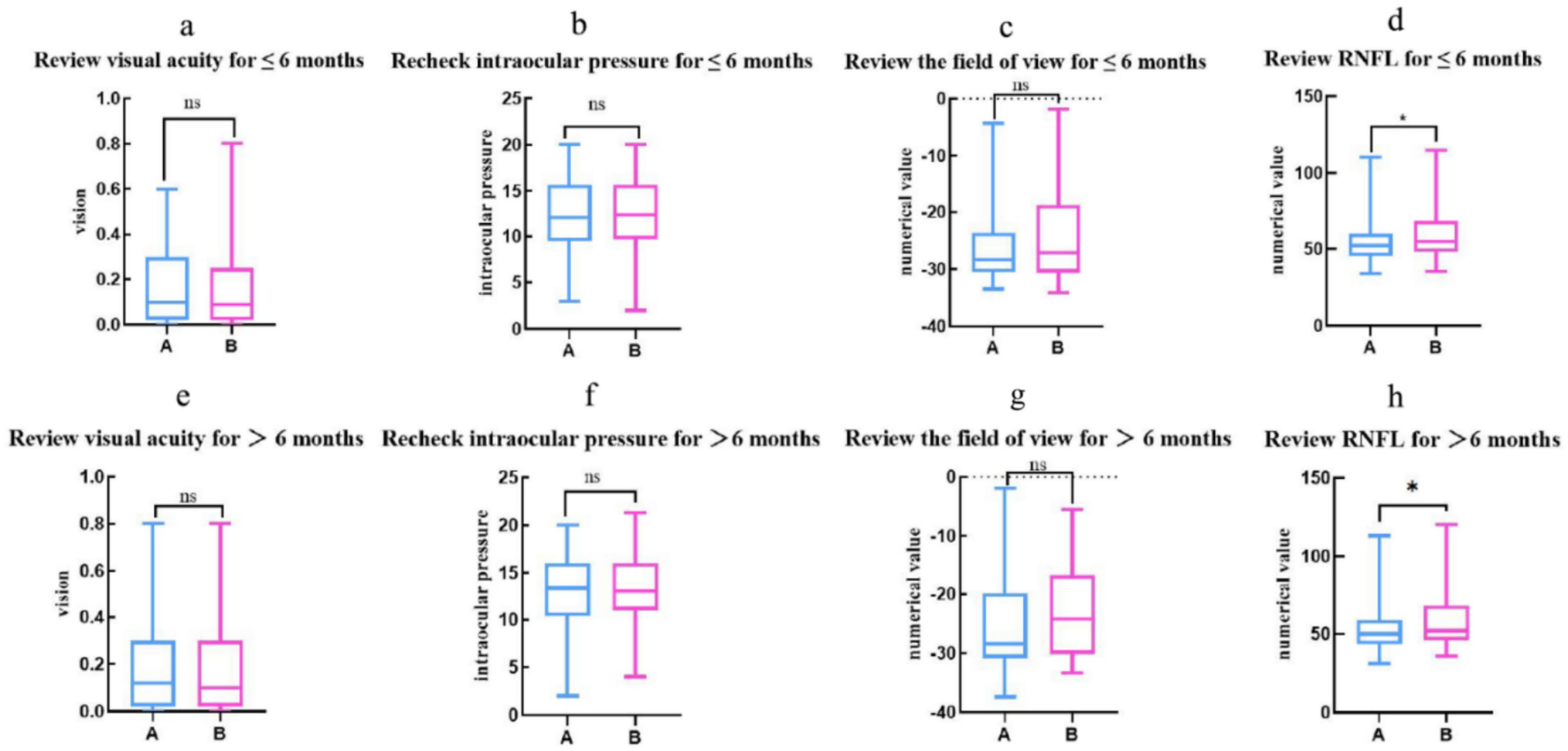
The two groups were re-examined up to 6 months postoperatively for visual acuity, intraocular pressure, and visual field (*p* > 0.05) as well as for RNFL (*p* < 0.05), with the X-axis representing the DRG group and None-DRG group, the Y-axis representing the numerical values of visual acuity. **(a,e)** The Y-axis represents the intraocular pressure (mmHg) value. **(b,f)** The Y-axis represents the numerical value (dB) of the field of view. **(c,g)** The Y-axis represents the RNFL (μm) state. **(d,h)** Statistical tests: **(a,c–h)** uses the Mann Whitney test, **(b)** uses the *T*-test; Significance level: ^*^ = *p* < 0.05;” ns” indicates that it is not significant.

For the clinical indicators of the two groups at ≤ 6 months after discharge and at re-examination > 6 months after discharge, the Mann Whitney U test was employed (Figure 4). The results indicated no statistically significant differences in visual acuity (*p* = 0.29), intraocular pressure (*p* = 0.98), and visual field (*p* = 0.09) between the groups at ≤ 6 months of re-examination. However, there was a statistically significant difference in the retinal nerve fiber layer (RNFL) thickness (*p* = 0.01 < 0.05) at ≤ 6 months of re-examination between the groups. Beyond 6 months of re-examination, there were no statistically significant differences in visual acuity (*p* = 0.89), intraocular pressure (*p* = 0.84), and visual field (*p* = 0.06) between the groups. However, the RNFL thickness remained statistically different (*p* = 0.04 < 0.05) between the groups at > 6 months of re-examination.

**Figure 5:**
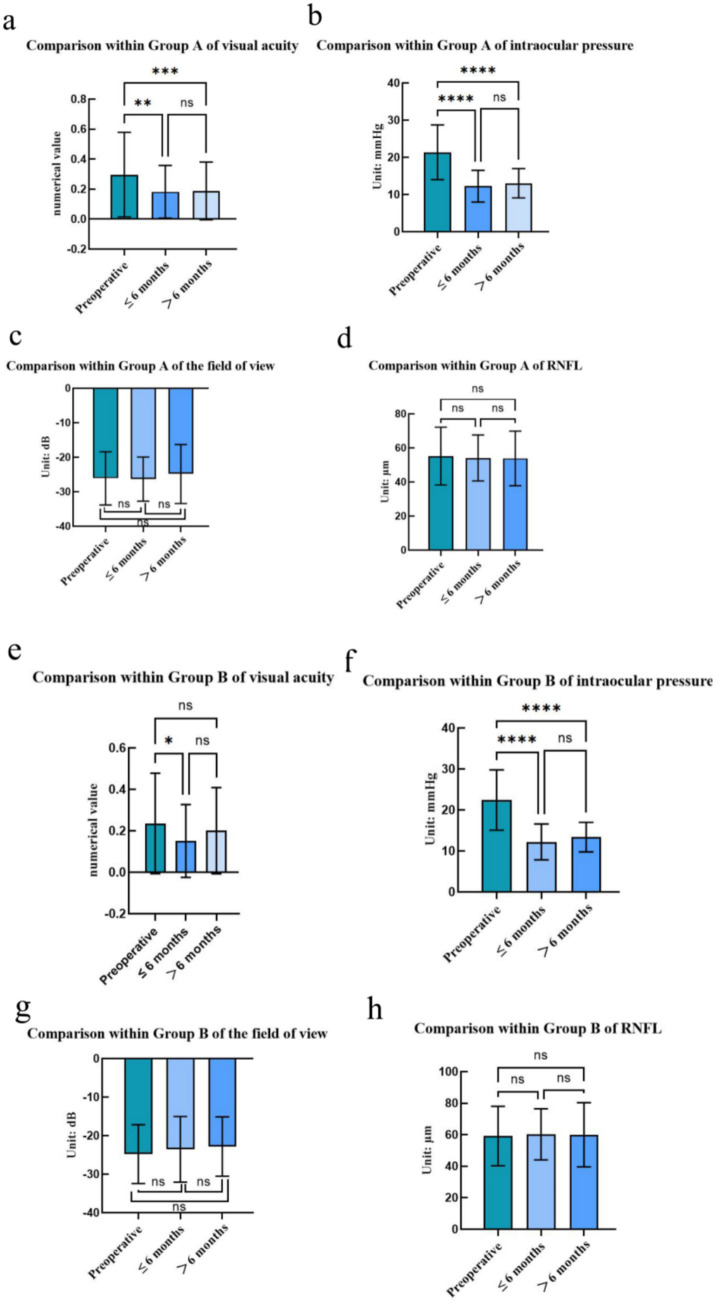
Comparison of visual acuity, intraocular pressure, visual field, and RNFL within DRG group, **(a–d)** Comparison of visual acuity, intraocular pressure, visual field, and RNFL within None-DRG group. **(e–h)** Statistical testing: one-way analysis of variance and Tukey multiple comparison *post hoc* test; Significance level: ^*^ = *p* < 0.05; ^**^ = *p* < 0.01; ^***^ = *p* < 0.001; ^****^ = *p* < 0.0001; “ns” represents no significant difference.

We compared the visual acuity, intraocular pressure, visual field, and RNFL of groups A and B within the group (Figure 5). The results of one-way *ANOVA* showed that in DRG group, the difference in variable visual acuity and intraocular pressure was statistically significant between preoperative and postoperative ≤6-month follow-up (*p* < 0.05), while the variable visual field and RNFL were not statistically significant. Compared with the 6-month follow-up > after surgery, there was a statistically significant difference in variable visual acuity and intraocular pressure (*p* < 0.05), while there was no significant difference in variable visual field and RNFL. There was no significant difference in variable visual acuity, intraocular pressure, visual field, and RNFL at 6 months ≤ and 6 months post>operative follow-up (*p* > 0.05). In None-DRG group, there were statistically significant differences in variable visual acuity and intraocular pressure between preoperative and postoperative ≤6-month follow-up (*p* < 0.05), while variable visual field and RNFL were not statistically significant, while the difference in variable intraocular pressure was statistically significant between preoperative and postoperative >6-month follow-up (*p* < 0.05), while variable visual acuity, visual field, and RNFL were not statistically significant. No statistically significant differences were observed in visual acuity, intraocular pressure, visual field, and RNFL between ≤ 6 months postoperatively and at postoperative >6-month follow-up (*p* > 0.05).

**Figure 6:**
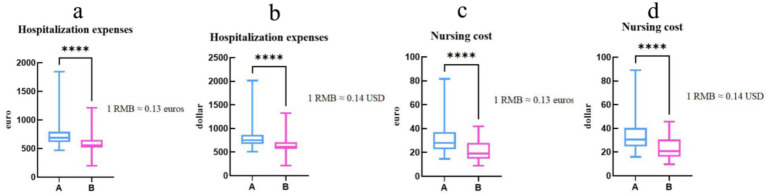
The hospitalization and nursing expenses for two groups of patients are expressed in US dollars (*p* < 0.001). **(a,b)** The hospitalization and nursing expenses of two groups of patients are expressed in euros (*p* < 0.0001). **(c,d)** In the bar chart, blue represents DRG group, pink represents None-DRG group, the X-axis represents Group, the Y-axis represents USD or EUR, Statistical test: Mann Whitney test; Significance level: ^* ** *^ = *p* < 0.001.

Finally, we compared the hospitalization and nursing expenses for patients in groups A and B (see Figure 6 for details). To facilitate cross-currency comparisons, we converted the RMB expenses into US dollars and euros using the January 2022 exchange rates: 1 RMB ≈ 0.14 US dollars, 1 RMB ≈ 0.13 euros. The hospitalization cost (USD) of group A was 143303.83$, group B was 82842.35$, group B decreased by 60461.48$ (*p* < 0.001) compared with group A, group A nursing cost (USD) was 5998.30$, group B was 2856.20$, and group B decreased by 3142.10$ (*p* < 0.001) compared with group A, with statistical differences. The cost of hospitalization and care for patients in group B was significantly lower than that of group A, both in terms of dollars and euros.

## Discussion

During our research period, the implementation of DRG system was associated with a statistically significant decrease in the average length of hospital stay. The average hospital stay decreased from 8 days (DRG group) to 5 days (non-DRG group). However, no statistically significant differences were observed in the postoperative follow-up measures (including visual acuity, intraocular pressure, and visual field) between the two groups at ≤ 6 months and > 6 months, as indicated by a *p*-values of more than 0.05. Additionally, no significant variations were observed in Retinal Nerve Fiber Layer (RNFL) measurements between the two groups during the same follow-up period. In comparison to DRG group, the cost of hospitalization and nursing care was significantly lower in None-DRG group, suggesting that the DRG model can effectively reduce the average length of hospital stay without directly impacting patient prognosis. Moreover, it can contribute to reducing hospitalization costs, assisting in medical insurance costs management, and reduce nursing costs. This finding supports the “quality-oriented” policy objective in China’s DRG payment system, suggesting that policymakers could: include glaucoma as a core DRG disease category and refine subgroup classification criteria; incorporate both “average length of hospital stay” and “complication incidence rate” into the performance evaluation system; promote collaboration between tertiary hospitals and community medical institutions to establish regional coordination mechanisms. Additionally, it is recommended that medical insurance departments expand the scope of DRG payment to cover more ophthalmic diseases.

Following the introduction of DRG, None-DRG group displayed a reduction in average hospital stay by 3 days in comparison to DRG group. This finding aligns with existing literature evidence from both domestic and international sources (6). The DRG grouping tool refines and decomposes the average length of stay management goal to departments, adopts the “benchmarking method” and combines CMI values, and transforms the average length of stay from outcome-based control to process-based control through the optimization and management of clinical pathways (7). The core of the DRG payment system is to enhance efficiency, improve medical quality, and control costs. Consequently, the centralized management approach that groups patients with similar clinical conditions together, along with the optimization of diagnostic and therapeutic service processes, can effectively shorten the average length of hospital stay, enhance bed utilization efficiency, and reduce time consumption.

In this study, no significant differences were found in visual acuity, intraocular pressure, and visual field between None-DRG group and DRG group at 6 months and beyond 6 months of follow-up under the DRG payment system compared to DRG group (*p* > 0.05). However, a statistically significant difference was observed in the retinal nerve fiber layer (RNFL) thickness between the two groups at preoperative and postoperative follow-up (≤ 6 months and > 6 months) (*p* < 0.01). This finding is supported by a prospective, observational, non-interventional study conducted by Schwenn, Oliver et al. (8). The irreversible damage to the optic nerve caused by glaucoma, and the permanent nature of neuronal cells, suggest that once neuronal cells die, they cannot regenerate. Consequently, RNFL thickness is not expected to change significantly over the duration of preoperative and postoperative follow-up. The underlying reason may be attributed to the fact that glaucoma, a blinding ocular disease primarily characterized by optic nerve damage, causes irreversible damage to the optic nerve. Neuronal cells, which constitute the fundamental units of the optic nerve, cannot regenerate once damaged or die. Consequently, the thickness of the retinal nerve fiber layer (RNFL), which represents the continuation of optic nerve fibers in the retina, remains relatively stable throughout the course of glaucoma and is less susceptible to significant changes over time. This characteristic was further corroborated in the present study, where RNFL values demonstrated no significant fluctuations during pre-operative and post-operative follow-ups (both ≤6 months and >6 months). Additionally, Ekici et al. (9), and colleagues conducted a prospective, longitudinal, observational cohort study to investigate the effects of glaucoma on quality of life and visual function over a 4-year period. Potential negative effects on visual acuity, intraocular pressure, visual field, and retinal nerve fiber layer (RNFL) in the absence of surgical treatment and active intervention. In our study, the clinical indicators of the 6-month follow-up ≤and > 6 months after the active intervention of composite trabeculectomy before and after the implementation of DRG showed good results, indicating that the roll-out of DRG payment system has a favorable impact on clinical outcomes. All clinical indicators of the patients exhibited a favorable recovery trend and stability. Consequently, there is sufficient rationale to believe that the promotion and application of the DRG payment system hold significant implications for enhancing the quality of ophthalmic medical services and improving patient outcomes in China, thereby warranting broader implementation and application.

In DRG group and None-DRG group, there was a statistically significant difference in visual acuity and intraocular pressure between preoperative and postoperative ≤6-month follow-up (*p* < 0.05), which aligns with the research conclusion of Ekici et al. (9) that, without surgical treatment and active intervention, visual acuity and intraocular pressure deteriorate. In our study, visual acuity and intraocular pressure improved ≤ 6 months after surgery, which may be attributed to the goal of composite trabeculectomy surgery, which is to maintain the patient’s existing vision, prevent further visual impairment, reduce intraocular pressure, slow down the progression of glaucoma, and prevent the deterioration of the disease. In DRG group and None-DRG group, there was a statistically significant difference in variable intraocular pressure between preoperative and postoperative >6-month follow-up (*p* < 0.05), which is consistent with the findings of Martin et al. (10) that persistent intraocular pressure elevation is a primary risk factor for glaucoma and a direct contributor to blindness. The purpose of surgery is to control intraocular pressure, explaining why intraocular pressure is more stable> 6 months postoperatively than before surgery.

The results of the study showed that there was a statistically significant difference in the hospitalization and nursing costs of patients in None-DRG group compared with DRG group after the implementation of the DRG payment system, which is consistent with the results of existing studies in this field (11), and the DRG payment system plays an important role in controlling medical costs and reducing the financial burden of patients. Under the DRG payment system, the average length of stay is reduced, which significantly affects the hospitalization costs and nursing costs of glaucoma patients. Specifically, the reduction in average length of hospital stay directly results in a decrease in various expenses incurred during patients’ hospitalization, including but not limited to bed charges, examination fees, treatment costs, and nursing expenses. This reduction in expenses not only saves patients a substantial amount of medical expenditure but also alleviates, to a certain extent, the economic burden on patients’ families. Studies in health economics and clinical practice have shown that unnecessary hospitalizations increase costs and have a negative impact on the economic well-being of hospitals paid for by DRG. Under traditional payment models, hospitals may, driven by economic incentives, tend to prolong patients’ hospital stays to generate higher medical revenue. However, in the DRG payment system, hospital reimbursement is based on predetermined groupings rather than the actual length of a patient’s stay. For glaucoma patients, shorter hospital stays not only reduce hospitalization and nursing costs but also mitigate the risk of hospital-acquired infections, thereby enhancing patients’ quality of life. Furthermore, the DRG payment system incentivizes hospitals to prioritize the improvement of medical quality and the optimization of healthcare services to meet DRG grouping criteria, fostering better development amidst fierce market competition.

Research has shown that, when applied to traditional bed-to-nurse ratios and work hour measurements, the introduction of DRG group evaluation indicators has significantly enhanced nursing quality (12). Specifically, DRG assessment indicators can conduct precise evaluation and classification of nursing needs based on multiple factors such as patients’ disease types, severity of illness, and treatment modalities. Through this scientific evaluation approach, hospitals can allocate nursing human resources more rationally, ensuring that each patient receives nursing services commensurate with their medical condition. The findings indicate that under the DRG payment system, nursing staff have proactively developed clinical nursing pathways, implemented multidisciplinary team management models, introduced the concept of patient group management, and enhanced the supervision of the first page of medical records, which has led to improved patient satisfaction, quality of nursing and patient safety (11). Specifically, a clinical nursing pathway is a standardized and regulated set of nursing procedures formulated based on the individual circumstances of patients. It delineates the key focuses and objectives of nursing work at each stage, thereby enhancing the systematicness and coherence of nursing practices. The multidisciplinary team management model breaks down the barriers between traditional departments, fostering communication and collaboration among professionals from diverse specialties and elevating the overall quality of medical services. The concept of patient cohort management involves grouping patients with similar medical conditions for centralized management. Through activities such as health lectures and rehabilitation guidance, this approach enhances patients’ self-management capabilities and health awareness. Moreover, strengthening the supervision of the front page of medical records ensures the accuracy and completeness of medical information, providing a reliable basis for the assessment and improvement of medical quality.

Following the implementation of DRG payment systems, there has been a notable reduction in average length of hospital stays for patients, an increase in patient turnover rates, and a rise in the number of admissions per unit time (13). However, this also presents challenges to nursing staff, as the shortened average length of stay in the hospital places a double burden on nurses, who must now meet the demand for centralized nursing services in a shorter period of time and manage the flow of people inside and outside the ward, thereby, increasing the complexity of nursing work and significantly increasing the nursing workload (14). In such circumstances, nurses are required not only to possess solid professional skills but also to demonstrate excellent time management abilities, communication and coordination skills, as well as adaptability, in order to cope with the increasingly complex and arduous nursing workload. Meanwhile, hospitals should also adopt corresponding measures, such as strengthening nurse training, optimizing nursing processes, and providing necessary support and resources, to assist nurses in better addressing these challenges and ensuring that nursing quality and patient safety remain uncompromised.

Our research has following strengths. Firstly, in line with the national development policy, a retrospective study was conducted to verify whether the DRG payment system would undermine the quality and safety of medical care and whether it was worth promoting. Secondly the study utilized medical records from ophthalmology hospitals in Yunnan Province, China, providing a regional perspective. Finally, this study illustrates the challenges and opportunities faced by caregivers under the DRG payment system.

Moving forward, our research group plans to utilize the medical alliance platform to incorporate the relevant data of medical institutions at all levels within the region for multi-center, prospective, large-sample, randomized controlled study. This will provide a stronger basis for reducing the average length of stay for patients with chronic diseases such as glaucoma. Future research can use the DRG payment system as a model and the average length of hospital stay as a mediator to explore the prognostic effect relationship of other diseases, thereby providing additional evidence for the promotion of the DRG payment system. Additionally, empirical research could explore how nursing staff navigate the opportunities and challenges presented by the DRG payment system, offering insights for enhancing the quality and safety of nursing care.

This study has several limitations: (1) Single-center design limits generalizability to broader populations; (2) Small sample size reduces statistical power and increases sampling error; (3) Wide age span complicates uniform assessment of factor impacts across age groups; (4) Potential gender imbalance, though baseline comparability was maintained, may affect external validity; and (5) Retrospective cohort design introduces confounding risks due to incomplete/inaccurate medical records. Future multi-center, large-sample studies are needed for more robust conclusions on ophthalmic disease factors.

## Conclusion

This study demonstrated that the implementation of the DRG medical insurance reform significantly curtailed hospitalization and nursing expenses by reducing the average length of stay for patients undergoing compound trabeculectomy from 8 days to 5 days. Notably, this cost reduction did not compromise prognostic indicators, such as postoperative visual acuity and intraocular pressure. These findings underscore the cost-effectiveness of DRG payment system within the realm of ophthalmology and furnish policymakers with empirical evidence to refine reimbursement strategies for ocular surgical procedures. Looking ahead, future investigations should delve deeper into the synergistic potential of integrating DRG with Enhanced Recovery After Surgery (ERAS) care pathways, aiming to concurrently enhance both medical quality and efficiency.
